# Complete mitogenome of the dog cucumber tapeworm *Dipylidium caninum* (Cestoda, Dilepididae) from Southwest China

**DOI:** 10.1080/23802359.2019.1644236

**Published:** 2019-07-22

**Authors:** Yue Xie, Yunjian Liu, Xiaobin Gu, Xiaduo Meng, Lu Wang, Yingxin Li, Xuan Zhou, Youle Zheng, Zhicai Zuo, Guangyou Yang

**Affiliations:** a Department of Parasitology, College of Veterinary Medicine, Sichuan Agricultural University, Chengdu, China;; b Institute of Animal Genetics and Breeding, College of Animal Science and Technology, Sichuan Agricultural University, Chengdu, China;; c Key Laboratory of Animal Disease and Human Health of Sichuan Province, College of Veterinary Medicine, Sichuan Agricultural University, Chengdu, China

**Keywords:** *Dipylidium caninum*, mitogenome, phylogeny

## Abstract

The cucumber tapeworm *Dipylidium caninum* (Cestoda, Dilepididae) is a common intestinal parasite of dogs and cats and can cause dipylidiasis in humans, especially in infants and children. In this study, the complete mitogenome of this tapeworm was sequenced using next-generation sequencing technology. The entire genome was 14,226 bp in size and encoded 36 genes, including 12 protein-coding genes, 22 transfer RNA genes, and 2 ribosomal RNA genes. The phylogeny revealed that *D. caninum* grouped with other species from the order Cyclophyllidea and separated from species of Pseudophyllidea. Within the Dipylidiidae, both dog-originated *D. caninum* were phylogenetic distinctiveness from cat-originated *D. caninum*, suggesting that *D. caninum* may represent a species complex. Altogether, the complete mitogenome of *D. caninum* sequenced here should contribute to a better understanding of the phylogenetic and taxonomic placement of this species.

*Dipylidium caninum* (Cestoda, Dilepididae) is a common intestinal cestode parasite of dogs and cats and can cause dipylidiasis in humans, especially in infants and children 1–5 years old (Chappell et al. 1990; Craig and Ito 2007; Jiang et al. 2017). This parasite is transmitted through the ingestion of the intermediate host fleas of dogs (*Ctenocephalides canis*) or cats (*Ctenocephalides felis*) which carry the larval forms (Craig and Ito 2007). Humans become infected by accidental ingestion of the infected fleas and in major cases, it mainly occurs in young children due to their playing habits and living in close proximity to infected dogs or cats (Chappell et al. 1990; García-Agudo et al. 2014; Jiang et al. 2017). Since first described in 1758, *D. caninum* infection or dipylidiasis has been recorded in at least 23 countries including USA, China, Japan, Italy, Chile, India, Mexico, Brazil, Canada, Sri Lanka, Uruguay, Argentine, Australia, Bulgaria, Cuba, Germany, Guatemala, Puerto Rico, Romania, South Africa, Spain, Turkey, and UK (Jiang et al. 2017). Current diagnosis of this zoonotic infection is typically based on morphological examination of rice-grain-like proglottids or egg packets in faeces (Craig and Ito 2007; Hogan and Schwenk 2019). However, morphological characteristics can often be unrecognized even by the experienced microscopists. Therefore, obtaining a more efficient and reliable approach to identify *D. caninum* infection has become crucial for clinical diagnosis and epidemiological investigation and achieving this goal is foreseeable only through utilization of molecular methodologies. Mitochondrial DNA (mtDNA) is regarded as an important and efficient source of molecular markers, being widely used for species-specific identification and differentiation of many zoonotic parasites (Le et al. 2002; Hu and Gasser 2006). Here, we reported the complete mitochondrial genome sequence of a representative *D. caninum* from China and further determined its phylogenetic and taxonomic placement with other related tapeworm species.

The parasite samples were obtained from an infected stray dog housed in an animal shelter at Wenjiang, Sichuan Province of Southwest China, after treatment with praziquantel. After morphological identification, all tapeworms (*n* = 4) were identified as *D. caninum* according to the taxonomic key of Venard (1937). One tapeworm used for DNA extraction was kept in 70% ethanol solution and the others were fixed in 5% formalin solution and archived in the Parasitological Museum of Sichuan Agricultural University (Sichuan, China) under collection numbers XY2018_1-3. Total mtDNA was isolated and sequenced using the Illumina HiSeq platform (Novogene, Tianjin, China). The mitogenome assembly was carried out with MITObim (Hahn et al. 2013) and gene annotation was performed by MITOS (Bernt et al. 2013).

The complete mitogenome of *D. caninum* was 14,226 bp in size (GenBank accession no. MN099047) with 72.9% AT and encoded 12 protein-coding genes, 22 tRNA genes, and 2 rRNA genes. All genes were unidirectionally transcribed on the plus strand, typical for other flatworms reported to date. Among the 12 protein-coding genes, except *cox3* and *nad2* deduced to use an incomplete stop codon ‘TA’, the rest were predicted to use the typical TAA or TAG as the stop codons. Twenty-two tRNA genes ranged from 59 bp (tRNA^(AGN)^-Ser) to 71 bp (tRNA-Ala) in size. Both 12S and 16S rRNAs were 635 bp and 968 bp in size, respectively, and located in the position between *cox2* and tRNA-Thr with a separation by tRNA-Cys. Two large non-coding regions, namely, NC1 (658 bp) and NC2 (212 bp), were placed between tRNA^(UCN)^-Ser and tRNA^(CUN)^-Leu and between *nad5* and tRNA-Gly, respectively, similar to other tapeworms, suggesting their conservation and function in regulation of transcription and control of DNA replication (Clayton 1991).

A maximum-likelihood (ML) phylogeny was reconstructed on a concatenated amino acid dataset of 12 protein-coding genes from 44 flatworms, using one trematode species *Schistosoma japonicum* as outgroup. As shown in Figure 1, this tree topology clearly placed *D. caninum* together with other species from the order Cyclophyllidea and separated from species of Pseudophyllidea with high bootstrap confidence, supporting that the Cyclophyllidea and Pseudophyllidea are monophyletic groups in the class Cestoda. Within the Cyclophyllidea clade, the families Taeniidae, Paruterinidae, Anoplocephalidae, Hymenolepididae, and Dipylidiidae further formed sub-monophyletic groups. Within the family Dipylidiidae, two dog-originated *D. caninum* (one was from China and another was from Japan) clustered together and then both were phylogenetic distinctiveness from cat-originated *D. caninum* (South Africa isolate), to some extent, suggesting that *D. caninum* may represent a species complex. In conclusion, the complete mitogenome of *D. caninum* deduced in this study provides essential DNA data for further phylogenetic and evolutionary analysis of this cucumber tapeworm.

**Figure 1.**
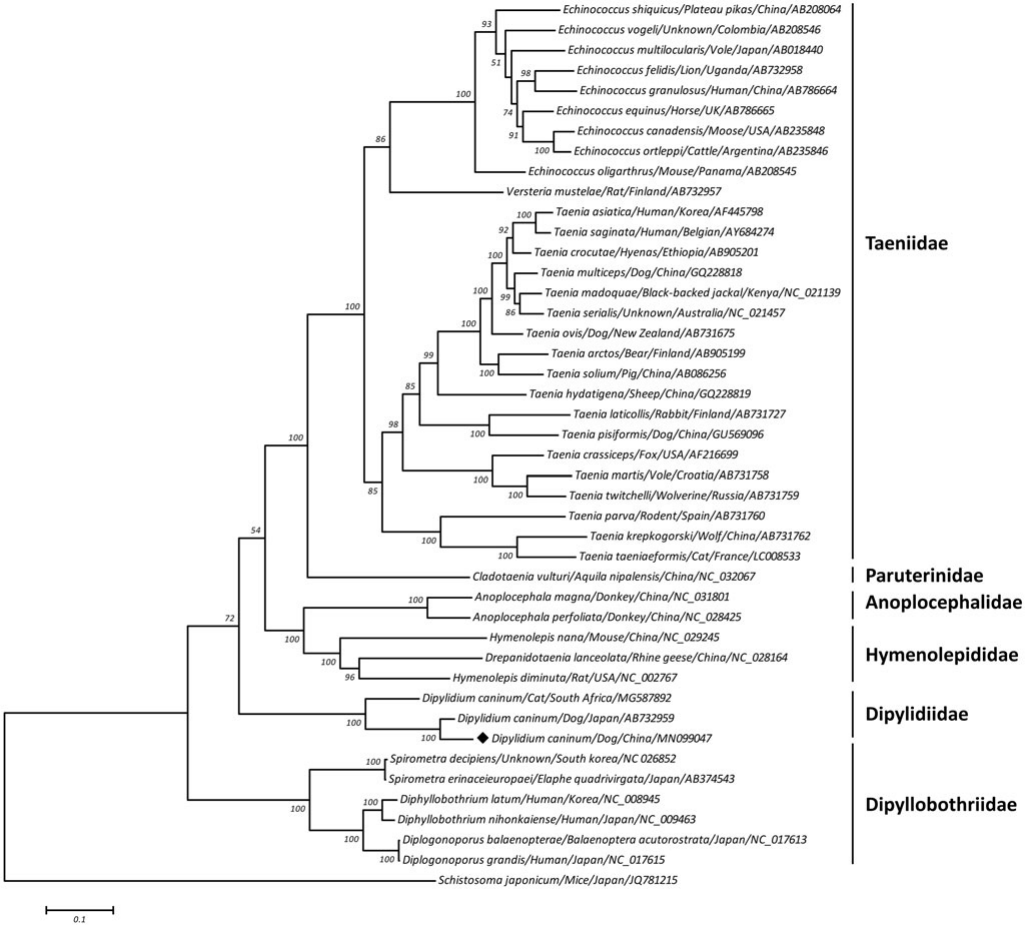
Maximum likelihood tree inferred from concatenated amino-acid sequences of 12 mt protein-coding genes of *D. caninum* and other related flatworms, utilizing MtArt + I+G model and after 1000 bootstrap replications (<50% support not shown). The black diamond sign represents the species in this study.
